# Cytochrome P450-Based Drug-Drug Interactions of Vonoprazan *In Vitro* and *In Vivo*

**DOI:** 10.3389/fphar.2020.00053

**Published:** 2020-02-14

**Authors:** Yiran Wang, Changxiong Wang, Shuanghu Wang, Quan Zhou, Dapeng Dai, Jihua Shi, Xue Xu, Qingfeng Luo

**Affiliations:** ^1^ Department of Gastroenterology, Beijing Hospital, National Center of Gerontology, Institute of Geriatric Medicine, Chinese Academy of Medical Sciences, Beijing, China; ^2^ Department of Gastroenterology, The Sixth Affiliated Hospital of Wenzhou Medical University, The People's Hospital of Lishui, Lishui, China; ^3^ The Laboratory of Clinical Pharmacy, The Sixth Affiliated Hospital of Wenzhou Medical University, The People's Hospital of Lishui, Lishui, China; ^4^ The Key Laboratory of Geriatrics, Beijing Institute of Geriatrics, Beijing Hospital, National Center of Gerontology, National Health Commission, Institute of Geriatric Medicine, Chinese Academy of Medical Sciences, Beijing, China

**Keywords:** cytochrome P450, drug-drug interactions, cocktails, vonoprazan, drug metabolism

## Abstract

**Background:**

Vonoprazan fumarate is a potassium-competitive acid blocker that was developed as a novel acid-suppressing drug for multiple indications. As a potential alternative to proton-pump inhibitors, the determination of the drug-drug interactions is vital for further applications. Probe drug cocktails are a type of rapid, economical, and efficient approach for evaluating cytochrome P450 enzyme activities. Since vonoprazan is metabolized partly by cytochrome P450, cocktails were used to study CYP-based drug-drug interactions.

**Methods:**

This study was conducted both *in vitro* and *in vivo*. In the *in vitro* study of rat liver microsomes, ultra-performance liquid chromatography coupled to tandem mass spectrometry was utilized to assess the reversible inhibition of cytochrome P450 by vonoprazan by determining the concentration of probe drugs (phenacetin, bupropion, tolbutamide, dextromethorphan, midazolam, chlorzoxazone). The differences in the levels of probe drugs between the rat groups with or without vonoprazan administration were also tested in the rats.

**Results:**

*In vitro* analysis revealed that the IC_50_ values of midazolam, tolbutamide, dextromethorphan, and bupropion in rat microsomes were 22.48, 18.34, 3.62, and 3.68 μM, respectively, while chlorzoxazone and phenacetin displayed no inhibition. *In vivo* analysis revealed that midazolam, bupropion, dextromethorphan, and tolbutamide showed significant (*P* < 0.05) differences in distinct pharmacokinetic parameters after vonoprazan administration, while those of chlorzoxazone and phenacetin were not significantly different.

**Conclusion:**

The *in vitro* and *in vivo* results indicated that vonoprazan can inhibit CYP3A4, CYP2C9, CYP2D6, and CYP2B6, suggesting that the coadministration of vonoprazan with cytochrome P450 substrates should be performed cautiously in clinical settings.

## Introduction

Vonoprazan fumarate (TAK-438) is a potassium-competitive acid blocker (P-CAB) that was developed as a novel acid-suppressing drug and launched in 2015 (Garnock-Jones, 2015). As a potential alternative to proton-pump inhibitors (PPIs), vonoprazan has been verified to be superior to conventional PPIs or even more effective in distinct gastric acid-related clinical indications. A phase III multicenter study demonstrated that there was no significant difference between vonoprazan and lansoprazole in treating erosive esophagitis (Xiao et al., 2020). In terms of preventing the recurrence of low-dose or long-term aspirin-associated ulcers, vonoprazan has proven to be as effective as lansoprazole (Kawai et al., 2018). The superior effect to rabeprazole was observed in healing artificial ulcer after endoscopic submucosal dissection (Yamasaki et al., 2018). Since PPIs play a vital role in helping to eradicate *Helicobacter pylori* infection, vonoprazan has been applied in similar trails and is considered to be a substitute for PPIs in resistant groups (O'Connor et al., 2019).

Additionally, regarding the pharmacokinetic profile, vonoprazan exhibits some advantages over PPIs e.g., it takes effect more quickly, suppresses acid secretory more potently, and exhibits better tolerability (Jenkins et al., 2015). Specifically, 20 mg of vonoprazan once daily equals 60 mg of omeprazole b.i.d., which is also equivalent to esomeprazole 40 mg b.i.d. (Graham and Tansel, 2018). It has been reported that vonoprazan is metabolized in two ways: the oxidative part by cytochrome P450 (CYP) enzyme isoforms and the nonoxidative part by sulfotransferase (SULT2A1) (Yamasaki et al., 2017).

Cytochrome P450 (CYP), which represents a diverse group of enzymes found in liver microsomes, is significantly indispensable in biological metabolism (Wilkinson, 2005) by metabolizing a large group of clinically used drugs (van Dyk et al., 2018). Because these enzymes can facilitate the elimination of various drugs, or modify their pharmacologic activities, the inhibition of these enzymes caused by drug coadministration or drug abuse can account for the increasing risk of adverse reactions (Vazquez, 2018). Human CYP includes 18 families and 44 subfamily members, which are categorized by amino acid similarities. Although the functions of genes in human cytochrome clusters contained differ from those of mice (Barzi et al., 2017), human CYP have their functional counterparts in mice, providing us with efficient approaches for further drug-drug interaction (DDI) studies.

Thus, probe drug cocktails were developed to evaluate CYP activities and the potential of DDIs (Frye et al., 1997). After years of modification, the use of these cocktails is now a rapid, economical, and efficient approach for evaluating different CYP enzymes simultaneously but independently (Rowland et al., 2016). To our best of our knowledge, no comprehensive DDI study of vonoprazan both *in vitro* and *in vivo* using the cocktails approach. Additionally, the existing results of vonoprazan DDIs were constrained to limited types of CYPs or were contradictory (Kagami and Furuta, 2018). Vonoprazan has not yet been launched in many countries, however, considering its effectiveness and safety, it has substantial potential to be widely utilized. Therefore, determining its DDIs will be beneficial for future clinical applications. Hence, in the present study, we explored the latent drug-drug interactions of vonoprazan. We chose phenacetin (CYP1A2), bupropion (CYP2B6), tolbutamide (CYP2C9), dextromethorphan (CYP2D6), midazolam (CYP3A), and chlorzoxazone (CYP2E1) as the core cocktail probe drugs, and ultrahigh-performance liquid chromatography coupled with triple quadrupole electrospray tandem mass spectrometry (UPLC-MS/MS) was performed to determine the results sensitively and reliably.

## Materials and Methods

### Chemicals and Reagents

Phenacetin, bupropion, tolbutamide, dextromethorphan, midazolam, chlorzoxazone, and the diazepam (all purity> 98%) that used as internal standards (ISs) were purchased from J&K Scientific Ltd. (Beijing, China). Vonoprazan was purchased from Beijing Sunflower Scientific Ltd. (Beijing, China). Hydroxybupropion and hydroxymidazolam were purchased from Sigma-Aldrich (St Louis, USA). Dextrorphan, hydroxytolbutamide, hydroxychlorzoxazone, and 4-acetamidophenol were purchased from Toronto Research Chemicals (Toronto, Canada). Reduced nicotinamide adenine dinucleotide phosphate (NADPH) was acquired from Roche Pharmaceuticals Ltd. (Basel, Switzerland). Ultra-pure water was produced by Milli-Q, a reagent-standard water purification system (Millipore, Bedford, USA). Acetonitrile and methanol that of high performance liquid chromatography (HPLC) grade were obtained from Merck Company (Darmstadt, Germany).

### Instrumentation and Analytical Conditions

**Figure 1:**
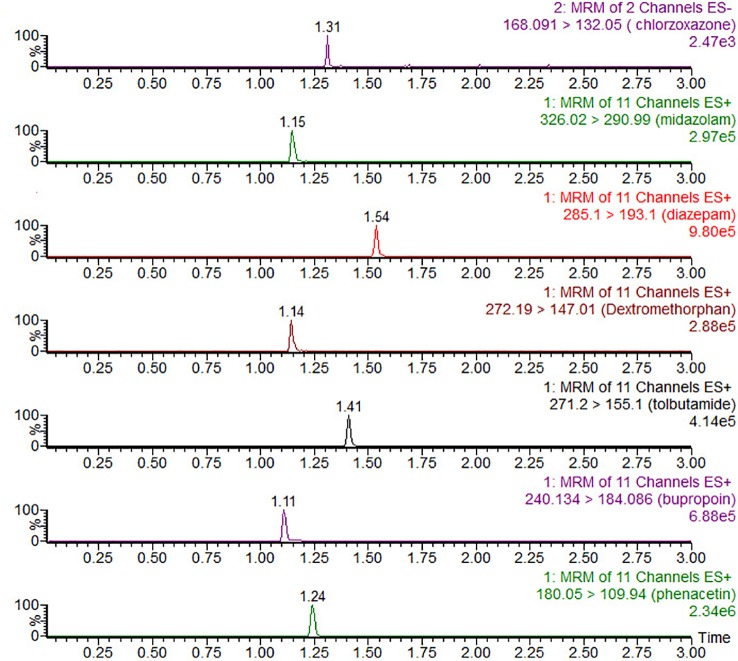
UPLC-MS/MS chromatograms, blank plasma spiked with chlorzoxazone, midazolam, diazepam (IS), dextromethorphan, tolbutamide, bupropion, and phenacetin.

The UPLC-MS/MS conditions were established as described previously (Ma et al., 2015) and were modified by adding chlorzoxazone and replacing metoprolol with dextromethorphan in the probe drug system. Chromatographic separation was performed using the Acquity UPLC system (Waters Corp., Milford, MA, USA) and Acquity BEH C18 column (2.1 mm × 50 mm, 1.7 μM) at 40°C. The mobile phase comprising acetonitrile (A) and 0.1% formic acid water (B) was set with a 0.40-ml/min flow rate. The gradient program was applied as follows: 0–0.6 min, 10–50% A; 0.6–1 min, 50–80%; 1–2 min, 80–95% A; 2–2.5 min, 95% A; 2.5–2.6 min, 95–10% A; 2.6–3 min, 10% A. XEVO TQD triple quadrupole mass spectrometer was equipped with electrospray ionization (ESI), and multiple-reaction monitoring (MRM) mode was selected for quantitation. The transitions are shown in **Table 1**. MassLynx 4.1 software (Waters Corp., Milford, MA, USA) was used for data acquisition, and the UPLC-MS/MS chromatogram of blank plasma that was spiked with probe drugs and metabolites is shown below (**Figure 1**).

**Table 1:** The transitions of probe drugs.

Compound	Parent	Daughter	Cone (V)	Collision (V)
Phenacetin	180.05	109.94	35	20
Bupropion	240.13	184.09	24	12
Tolbutamide	271.2	155.1	30	15
Dextromethorphan	272.19	147.01	45	30
Diazepam	285.1	193.1	35	30
Midazolam	326.02	290.99	50	28
Chlorzoxazone	168.09	132.05	48	20

### Preparation of Rat Liver Microsomes

The pooled rat liver microsomes (RLMs) obtained from eight rats, which were weighed and homogenized with cold 0.01 mM phosphate-buffered saline (PBS), containing 0.25 mM sucrose. After centrifugation for 15 min at 11,000 rpm, the supernatants were separated and transferred into new tubes for another 15-min centrifugation at 11,000 rpm. Next, ultracentrifugation was performed at 10,000 rpm at 4°C for 1 h., and the microsomal pellets were resuspended with cold 0.01 mM PBS and stored at −80°C (Wang et al., 2015). The protein concentration of RLMs was determined using the Bradford Protein Assay Kit (Thermo Scientific, Waltham, MA, USA).

### *In Vitro* Interaction Studies in Rat Liver Microsomes

Next, vonoprazan was applied as the inhibitor to determine the half maximal inhibitory concentration (IC50). The incubation mixture in a total volume of 200 μl contained 0.5 mg/ml of RLMs, 1 mM potassium phosphate buffer (pH 7.4), the probe drug mixture that dissolved by dimethyl sulfoxide (40, 20, 10, 100, 20, and 5 μM for phenacetin, bupropion, tolbutamide, dextromethorphan, chlorzoxazone, and midazolam, respectively), vonoprazan (1, 2.5, 5, 10, 25, 50, and 100 μM) and 1 mM NADPH (Stoetzer et al., 2015). After preincubation in a shaking water bath at 37°C for 5 min, an NADPH-regenerating system was added to initiate the reaction in a final volume of 200 μl. The reaction was performed for 30 min, and was stopped by cooling to −80°C immediately. Next, 20 μl of internal standard (IS) working solution and 200 μl of acetonitrile were added. Centrifugation was performed at 13,000 rpm for 5 min after vortexing for 1 min. The supernatant mixture (2 μl) was injected into the UPLC–MS/MS system for analysis.

### Pharmacokinetic Study of Vonoprazan

Ten specific pathogen-free (SPF) grade Sprague–Dawley rats (male, 220 ± 20 g) were provided by Wenzhou Medical University Laboratory Animal Research Center. This study was carried out in accordance with the principles of the Basel Declaration and recommendations of Wenzhou Medical University Administration Committee of Experimental Animals. The protocol was approved by the Wenzhou Medical University Administration Committee of Experimental Animals (ID Number: wydw2019-650). Ten rats were randomly divided into two groups: the vonoprazan group (n = 5) and control group (n = 5). Both groups were fasted overnight before experiments but were allowed water all the time. The six probe drug mixtures and vonoprazan were dissolved in 0.5% carboxy methyl cellulose sodium (CMC-Na), respectively. The vonoprazan group was administered 5 mg/kg of vonoprazan for 14 days. The control group was administered 0.5% CMC-Na. After 30 min of vonoprazan or 0.5% CMC-Na, on the 14^th^ day, the two groups were treated by gavage at a single dosage of 10 mg/kg for bupropion, dextromethorphan, phenacetin, midazolam, and chlorzoxazone, and of 1 mg/kg for tolbutamide.

### Sample Collection and Preparation

Blood samples (300 µl) were collected *via* the tail vein at the time points of 0.083, 0.25, 0.5, 1, 2, 3, 4, 6, 8, 12, and 24 h. after gavage. Next, the samples were collected into 1.5-ml centrifuge tubes and were immediately centrifuged for 10 min at 4,000 rpm speed. For every 100 µl of rat plasma was added with 200 µl of acetonitrile in 0.5 μg/ml of IS was added, followed by vortex mixing for one minute. After centrifugation at 13,000 rpm for 15 min, 5 µl of supernatant was prepared for UPLC-MS/MS system to analysis. *In vitro* DDI detection system, the pH was 7.4 and the component of buffer was consisted of 100 mM potassium phosphate.

### Statistical Analysis

The GraphPad (version 7.0; GraphPad Software Inc., San Diego, CA, USA) was applied to calculate IC50 values and plot plasma concentration-time curves. The pharmacokinetic parameters using noncompartmental analysis was calculated by DAS (version 3.2.8; Wenzhou Medical University, China). Statistical comparisons within groups were conducted by SPSS (version 25.0; SPSS Inc., Chicago, IL, USA), using Student's t-test. A *P*-value <0.05 was considered statistically significant.

## Results

### Effects of Vonoprazan on the Metabolism of Probe Drugs *In Vitro*

**Figure 2:**
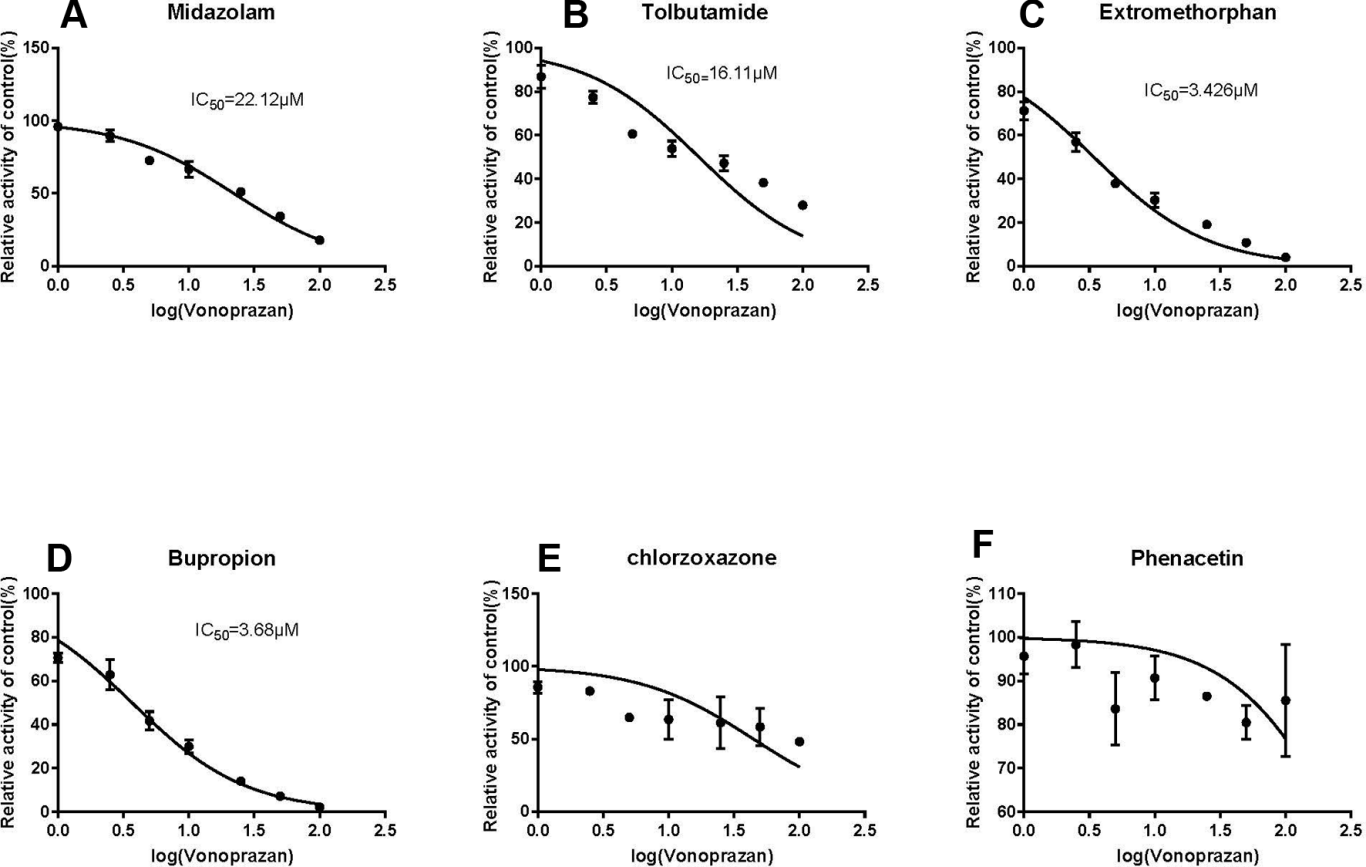
The inhibitory effect of vonoprazan on midazolam **(A)**, tolbutamide **(B)**, dextromethorphan **(C)**, bupropion **(D)**, chloraoxazone**(E)**, and phenacetin **(F)** for IC50 values in RLMs (values are Mean ± SD, n = 3).

Following vonoprazan addition, the metabolism of the four probe drugs midazolam, tolbutamide, dextromethorphan, and bupropion were inhibited to various degrees (**Figure 2**). Their IC_50_ values in rat microsomes were 22.48 µM (**Figure 2A**), 18.34 µM (**Figure 2B**), 3.62 Mm (**Figure 2C**), and 3.68 µM (**Figure 2D**), respectively. By contrast, chlorzoxazone (**Figure 2E**) and phenacetin (**Figure 2F**) displayed no inhibition. The results illustrated that vonoprazan can inhibit the metabolism of midazolam, tolbutamide, dextromethorphan, and bupropion in rat microsomes.

### Effects of Vonoprazan on the Metabolism of Probe Drugs *In Vivo*

**Figure 3:**
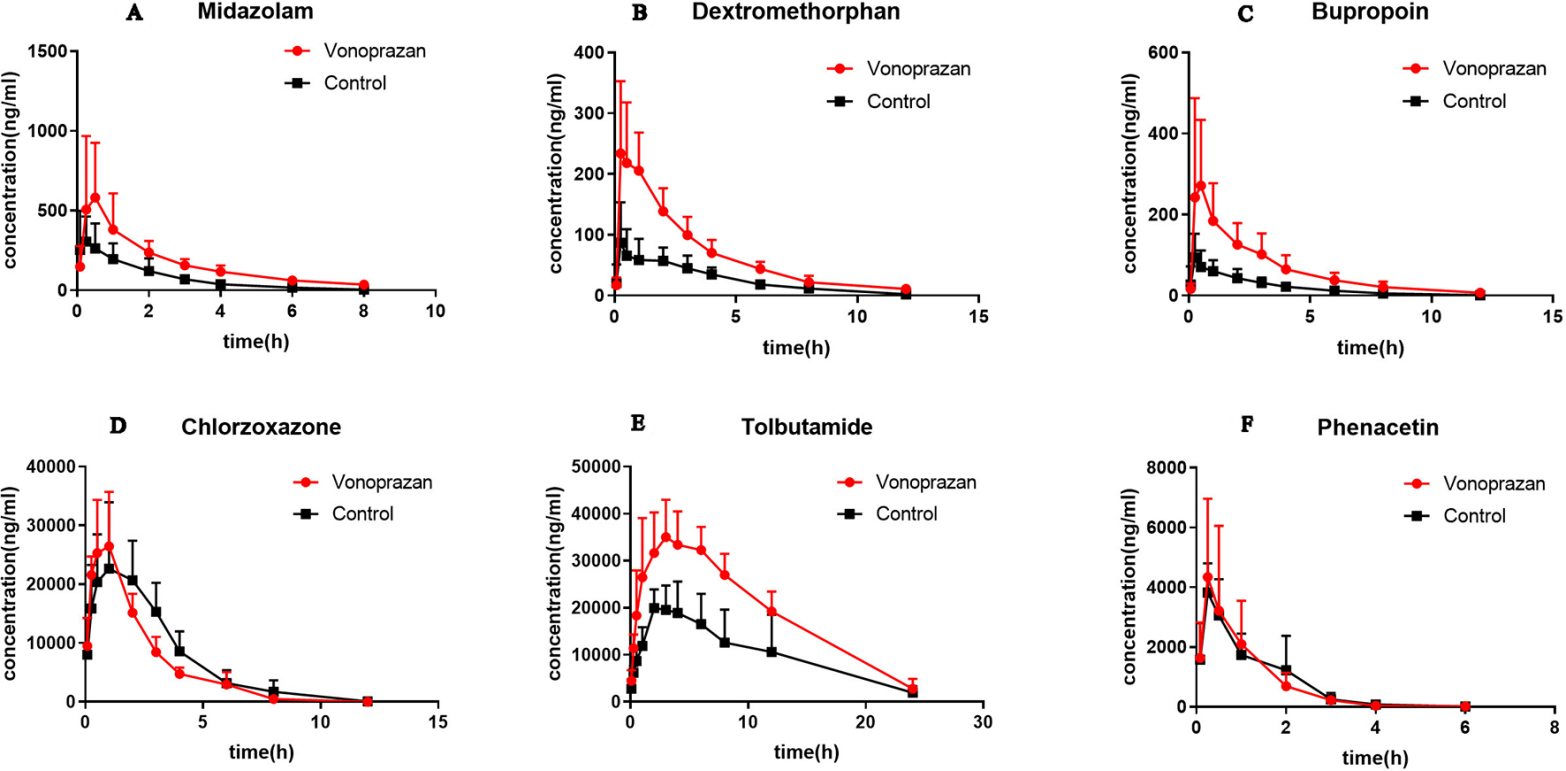
The pharmacokinetic profiles of midazolam **(A)**, dextromethorphan **(B)**, bupropion **(C)**, chlorzoxazone **(D)**, tolbutamide **(E)**, and phenacetin **(F)** in control group and vonoprazan group rats (n = 5).

The statistical analysis results of the mean pharmacokinetic parameters are demonstrated in **Tables 2**–**4**, which were analyzed by DSA 3.2.8. The mean plasma concentration *versus* time curves of each probe drug in the vonoprazan and control groups are presented in **Figure 3**.

**Table 2:** Pharmacokinetic parameters of probe drugs (midazolam and bupropion) from control group and vonoprazan group rats (mean ± SD, n = 5).

Parameters	Unit	Midazolam		Bupropion	
		Vonoprazan + midazolam	Midazolam	Vonoprazan+ bupropion	Bupropion
AUC _(0-t)_	ug/L*h	1,355.69 ± 539.80*	625.40 ± 194.16	767.76 ± 374.15*	241.87 ± 105.87
AUC _(0-∞)_	ug/L*h	1,480.79 ± 577.29*	656.94 ± 198.31	804.11 ± 384.27*	243.45 ± 106.60
MRT _(0-t)_	h	2.35 ± 0.39	1.90 ± 0.60	2.93 ± 0.59	2.72 ± 0.51
MRT _(0-∞)_	h	3.11 ± 0.68	2.34 ± 1.12	3.49 ± 1.33	2.78 ± 0.57
t_1/2z_	h	2.31 ± 0.48	1.85 ± 1.18	2.43 ± 1.13	1.38 ± 0.25
Tmax	h	0.45 ± 0.11*	0.18 ± 0.09	0.40 ± 0.14	0.37 ± 0.36
Vz/F	L/kg	24.50 ± 7.07	39.36 ± 20.44	48.17 ± 19.58	99.13 ± 53.70
CLz/F	L/h/kg	7.65 ± 2.95*	16.90 ± 7.15	15.24 ± 7.66*	51.79 ± 32.61
Cmax	ug/L	622.13 ± 420.88	335.42 ± 190.55	323.44 ± 197.10*	97.08 ± 55.54

*P < 0.05 indicates statistical difference between the two groups.

**Table 3:** Pharmacokinetic parameters of probe drugs (dextromethorphan and tolbutamide) from control group and vonoprazan group rats (mean ± SD, n = 5).

Parameters	Unit	Dextromethorphan		Tolbutamide	
		Vonoprazan + dextromethorphan	Dextromethorphan	Vonoprazan + tolbutamide	Tolbutamide
AUC _(0-t)_	ug/L*h	804.16 ± 205.63*	318.68 ± 107.33	462,333 ± 103,203*	248,799 ± 122,431
AUC _(0-∞)_	ug/L*h	842.68 ± 199.30*	336.61 ± 95.30	484,776 ± 126,240*	264,335 ± 138,164
MRT _(0-t)_	h	3.14 ± 0.47	3.47 ± 0.65	7.92 ± 0.29	7.65 ± 1.24
MRT _(0-∞)_	h	3.77 ± 1.04	4.35 ± 1.99	8.88 ± 1.04	8.72 ± 2.10
t_1/2z_	h	2.57 ± 0.94	2.61 ± 1.47	4.83 ± 1.27	4.99 ± 1.56
Tmax	h	0.45 ± 0.33	0.75 ± 0.77	3.60 ± 1.34	3.00 ± 1.00
Vz/F	L/kg	46.04 ± 19.59	125.47 ± 88.46	0.0140 ± 0.0020*	0.03 ± 0.01
CLz/F	L/h/kg	12.44 ± 3.12*	31.91 ± 9.87	0.002 ± 0.001	0.01 ± 0.00
Cmax	ug/L	275.76 ± 63.90*	95.96 ± 55.77	36,407 ± 6,755*	21,791 ± 3,736

*P < 0.05 indicates statistical difference between the two groups.

**Table 4:** Pharmacokinetic parameters of probe drugs (chlorzoxazone and phenacetin) from control group and vonoprazan group rats (mean ± SD, n = 5).

Parameters	Unit	Chlorzoxazone		Phenacetin	
		Vonoprazan + chlorzoxazone	Chlorzoxazone	Vonoprazan + phenacetin	Phenacetin
AUC _(0-t)_	ug/L*h	73,080.1 ± 12,891.2	89,528.9 ± 10,455.3	4,873.1 ± 2,596.0	5,037.1 ± 2,108.7
AUC _(0-∞)_	ug/L*h	73,138.9 ± 12,884.3	89,629.8 ± 10,476.8	4,891.2 ± 2,581.9	5,043.6 ± 2,107.0
MRT _(0-t)_	h	2.16 ± 0.37	2.69 ± 0.79	1.06 ± 0.20	1.12 ± 0.20
MRT _(0-∞)_	h	2.17 ± 0.37	2.70 ± 0.80	1.10 ± 0.25	1.13 ± 0.21
t_1/2z_	h	0.93 ± 0.33	0.93 ± 0.28	0.68 ± 0.31	0.60 ± 0.06
Tmax	h	0.75 ± 0.35	1.50 ± 1.00	0.30 ± 0.11	0.30 ± 0.11
Vz/F	L/kg	0.19 ± 0.08	0.15 ± 0.05	2.86 ± 2.41	1.97 ± 0.81
CLz/F	L/h/kg	0.14 ± 0.03	0.11 ± 0.01	2.65 ± 1.52	2.25 ± 0.80
Cmax	ug/L	27,449.6 ± 8,245.7	28,500.0 ± 5,519.9	4,798.2 ± 3,104.8	3,865.3 ± 1,020.4

Midazolam, bupropion, dextromethorphan, and tolbutamide showed significant differences (*P* < 0.05) in distinct pharmacokinetic parameters compared with the control group, while the parameters of chlorzoxazone and phenacetin were not significantly different. The AUC_0→t_ values of midazolam, bupropion, dextromethorphan, and tolbutamide were all increased greatly, by 1.17-fold, 2.17-fold, 1.52-fold, and 0.86-fold, respectively, compared with that of the control group. Additionally, the AUC_0→∞_ values were elevated by 1.25-fold, 2.30-fold, 1.50-fold, and 0.83-fold, respectively. Specifically, the addition of vonoprazan significantly increased the Tmax of midazolam and the Cmax values of bupropion, dextromethorphan, and tolbutamide by 1.50-fold, 2.33-fold, 1.87-fold, and 0.67-fold, respectively. Additionally, vonoprazan significantly decreased the CLz/F of midazolam, bupropion, dextromethorphan, and the Vz/F of bupropion by 54.7, 70.6, 61.0, and 53.3%, respectively.

These results indicate that vonoprazan has an inhibitory effect on the metabolism of midazolam, bupropion, dextromethorphan, and tolbutamide in rats, agreeing with the results obtained *in vitro*.

## Discussion

Vonoprazan, a P-CAB drug, was approved for application in the treatment of acid-related diseases in Japan on December 26, 2014 (Takeda, 2014). Previously, proton pump inhibitors (PPIs) were considered the first-in-class available drug to treat acid-related diseases, such as gastroesophageal reflux disease and gastroduodenal ulcers (Spechler et al., 2019), and has been widely utilized in the eradication therapy of *H. pylori* (Crowe, 2019). The unmet clinical needs of PPIs promote new drug development. Because the high acidic environment in the stomach is established by the gastric H+ and K+-ATPase(Yamamoto et al., 2019), classical PPIs have been produced previously. P-CABs can both block the H+ and K+-ATPase enzyme and reversibly bind K+ ion (Abe et al., 2018), enabling vonoprazan to show long duration and good bioavailability that can be less affected by the pH in the stomach (Hori et al., 2011). The better are the physicochemical characteristics, the more frequent are well-designed controlled clinical trials conducted. Thus, vonoprazan has been gradually applied in triple or quadruple regimens as a substitute for PPIs to enhance the antimicrobial effect against *H. pylori* because drug resistance to classical strategies has increased annually (Fallone et al., 2019). Vonoprazan was confirmed to be superior in the secondary prevention of nonsteroidal anti-inflammatory drug-induced peptic ulcers (Mizokami et al., 2018). Vonoprazan is also thought to potentially show superiority over PPIs in patients who are difficult to treat, given that it can reliably achieve a therapeutically required intragastric pH (Tansel and Graham, 2017).

Concerning the pharmacodynamics of vonoprazan, before it entered the market, *in vitro* studies demonstrated that it is mainly metabolized by CYP3A4 and partially by CYP2C19, CYP2B6, CYP2D6, and SULT2A1 (Echizen, 2016). At that time, its inhibitory effect on any CYP isozymes were considered would not to emerge in clinical settings. However, though the distinct CYP2C19 genotypes showed no appreciable correlation with its metabolism in initial study (Kagami et al., 2016), subsequent trials exhibited dissimilar results (Funakoshi et al., 2019), indicating there may be other possible DDIs. Thus, intending to explore as much DDIs as possible, we chose those six probe drugs for wider coverage: phenacetin (CYP1A2), bupropion (CYP2B6), tolbutamide (CYP2C9), dextromethorphan (CYP2D6), midazolam (CYP3A), and chlorzoxazone (CYP2E1). Those similar enzymes also have been detected in other clinical trials (Gravel et al., 2019). We utilized the system we established before, which is more convincing and reliable. But as rats do not have CYP2C19, the results are limited and it should be paid attention to this, if the trials on human were to be conducted.

This study investigated the potential DDIs *in vitro* by using rat liver microsomes and *in vivo* using performing ultra-performance liquid chromatography-tandem mass spectrometry to detect the metabolites of probe drugs in rat plasma. The *in vitro* results indicated that vonoprazan has direct inhibitory effects on CYP3A4 (IC_50_ = 22.48 µM), CYP2C9 (IC_50_ = 18.34 µM), CYP2D6 (IC_50_ = 3.62 µM), and CYP2B6 (IC_50_ = 3.68 µM), but no effect on CYP1A2 and CYP2E1. These findings partially confirm the results produced using making use of human liver microsomes (HLMs) (Nishihara et al., 2019). Nevertheless, the IC_50_ values in previous studies *via* HLMs were much higher than the maximum plasma concentrations of vonoprazan after therapeutic oral doses; therefore, theoretically, few clinical restraint effects should be considered. However, actual situations are more complex than expected. A previous study reported a reference maximum plasma concentration level in rats of 17 ng/ml (Kogame et al., 2017), indicating that the threshold value of vonoprazan could be reached and that the metabolism of these four probe drugs could be extended in rats *in vivo*.

Therefore, to further consolidate our results, pharmacokinetic interaction studies in rats *in vivo* were implemented. The main pharmacokinetic parameters (AUC0→t and AUC0→∞) of midazolam, bupropion, dextromethorphan, and tolbutamide were elevated significantly following vonoprazan, indicating vonoprazan indeed constrained their metabolism. Specifically, the oral clearance (CLz/F) of midazolam was decreased while the Tmax was increased, indicating vonoprazan affected midazolam by postponing the exposure and reducing the clearance. Regarding bupropion and dextromethorphan, vonoprazan may lessen the clearance and resulted in a rise in the maximum plasma concentration (Cmax). Regarding tolbutamide, the apparent volume of distribution (Vz/F) declined, implying that the vonoprazan approximately can enhance its binding ability, thus increasing Cmax. Similar to the *in vitro* findings, chlorzoxazone and phenacetin was not affected by vonoprazan. Because the diverse probe drugs represent corresponding CYP enzymes, considering all of the data, the results *in vitro* and *in vivo* were coherent: the vonoprazan can inhibit CYP3A4, CYP2C9, CYP2D6, and CYP2B6 to a distinct extent.

Drug interactions of PPI have been studied a lot and results are quite specific (Xie et al., 2019). As a feasible substitute in future, the vonoprazan's DDIs deserve explorations. Thus far, the DDI investigation of vonoprazan mainly concerned CYP3A4 and CYP2C19, with unconvincing consequences. In the triple regimens to eradicate H. pylori infection, whether the coadministration of vonoprazan and clarithromycin (CYP3A4 inhibitor) would have a mutual effect remains debatable (Jenkins et al., 2017; Sugimoto and Yamaoka, 2018). Additionally, Kagami et al. (2018) observed that vonoprazan could attenuate the function of clopidogrel, not depending on the CYP2C19 or CYP3A4 genotypes. Other CYP types were also confirmed to not be associated with reducing the antiplatelet effectiveness of both clopidogrel (Nishihara and Czerniak, 2018) and prasugrel (Nishihara, 2019). It seems the relationship between anti-acid and antiplatelet agents is more intricate. The controversial findings could be due to the heterogeneity of different studies or influenced by other type enzymes noted in this study. The potential inhibitory effects of vonoprazan on CYP2C9, CYP2D6, and CYP2B6 have been revealed in our results but didn't be paid much attention in other studies. Considering various common drugs are metabolized by these pathways: warfarin by 3A4 and 2C9 (Vazquez, 2018), tricyclic antidepressants by 2D6 (Gaedigk et al., 2017), nicotine by 2B6 (Tomaz et al., 2019), it has significant value to verify the authentic DDIs in clinical settings.

Owing to limited condition, we have not applied it to human beings yet. But it is necessary for further study and we will consider to perform it in future, basing on the present results that we get from rats. Furthermore, other inhibitory mechanisms can be explored (Yasumuro et al., 2018). Thus, stricter clinical trials will be needed to further confirm these findings.

In summary, our study investigated CYP-based drug-drug interactions both *in vitro* and *in vivo*. The results demonstrated that the vonoprazan could inhibit CYP3A4, CYP2C9, CYP2D6, and CYP2B6, suggesting that the coadministration of vonoprazan and CYP substrates should be performed cautiously in clinical settings.

## Data Availability Statement

All datasets generated for this study are included in the article/supplementary material.

## Ethics Statement

The animal study was reviewed and approved by the Wenzhou Medical University Administration Committee of Experimental Animals.

## Author Contributions

YW, CW, SW, and QZ contributed conception and design of the study. DD performed the statistical analysis. YW and CW wrote the first draft of the manuscript. SW, QZ, DD, JS, and XX wrote sections of the manuscript. All authors contributed to manuscript revision, read, and approved the submitted version. QL are accountable for all aspects of the work in ensuring that questions related to the accuracy or integrity of any part of the work are appropriately investigated and resolved.

## Funding

This research was funded by 135 Major National Science and Technology Projects (No. 2017ZX09304026).

## Conflict of Interest

The authors declare that the research was conducted in the absence of any commercial or financial relationships that could be construed as a potential conflict of interest.

## References

[B1] Abe K. Irie K. Nakanishi H. Suzuki H. Fujiyoshi Y. (2018). Crystal structures of the gastric proton pump. Nature 556 (7700), 214–218. 10.1038/s41586-018-0003-8 29618813

[B2] Barzi M. Pankowicz F. P. Zorman B. Liu X. Legras X. Yang D. (2017). A novel humanized mouse lacking murine P450 oxidoreductase for studying human drug metabolism. Nat. Commun. 8 (1), 39. 10.1038/s41467-017-00049-x 28659616 PMC5489481

[B3] Crowe S. E. (2019). Helicobacter pylori infection. N. Engl. J. Med. 380 (12), 1158–1165. 10.1056/NEJMcp1710945 30893536

[B4] Echizen H. (2016). The first-in-class potassium-competitive acid blocker, vonoprazan fumarate: pharmacokinetic and pharmacodynamic considerations. Clin. Pharmacokinet 55 (4), 409–418. 10.1007/s40262-015-0326-7 26369775

[B5] Fallone C. A. Moss S. F. Malfertheiner P. (2019). Reconciliation of recent helicobacter pylori treatment guidelines in a time of increasing resistance to antibiotics. Gastroenterology 157 (1), 44–53. 10.1053/j.gastro.2019.04.011 30998990

[B6] Frye R. F. Matzke G. R. Adedoyin A. Porter J. A. R.A. B. (1997). Validation of the five-drug “Pittsburgh cocktail” approach for assessment of selective regulation of drug-metabolizing enzymes. Clin. Pharmacol. Ther. 62 (4), 365–376. 10.1016/S0009-9236(97)90114-4 9357387

[B7] Funakoshi R. Tomoda Y. Kudo T. Furihata K. Kusuhara H. Ito K. (2019). Effects of proton pump inhibitors, esomeprazole and vonoprazan, on the disposition of proguanil, a CYP2C19 substrate, in healthy volunteers. Br. J. Clin. Pharmacol. 85 (7), 1454–1463. 10.1111/bcp.13914 30845361 PMC6595331

[B8] Gaedigk A. Sangkuhl K. Whirl-Carrillo M. Klein T. Leeder J. S. (2017). Prediction of CYP2D6 phenotype from genotype across world populations. Genet. Med. 19 (1), 69–76. 10.1038/gim.2016.80 27388693 PMC5292679

[B9] Garnock-Jones K. P. (2015). Vonoprazan: first global approval. Drugs 75 (4), 439–443. 10.1007/s40265-015-0368-z 25744862

[B10] Graham D. Y. Tansel A. (2018). Interchangeable use of proton pump inhibitors based on relative potency. Clin. Gastroenterol. Hepatol. 16 (6), 800–808.e807. 10.1016/j.cgh.2017.09.033 28964908 PMC6913203

[B11] Gravel S. Chiasson J. L. Turgeon J. Grangeon A. Michaud V. (2019). Modulation of CYP450 activities in patients with type 2 diabetes. Clin. Pharmacol. Ther. 106 (6), 1280–1289. 10.1002/cpt.1496 31099895

[B12] Hori Y. Matsukawa J. Takeuchi T. Nishida H. Kajino M. Inatomi N. (2011). A study comparing the antisecretory effect of TAK-438, a novel potassium-competitive acid blocker, with lansoprazole in animals. J. Pharmacol. Exp. Ther. 337 (3), 797–804. 10.1124/jpet.111.179556 21411494

[B13] Jenkins H. Sakurai Y. Nishimura A. Okamoto H. Hibberd M. Jenkins R. (2015). Randomised clinical trial: safety, tolerability, pharmacokinetics and pharmacodynamics of repeated doses of TAK-438 (vonoprazan), a novel potassium-competitive acid blocker, in healthy male subjects. Aliment Pharmacol. Ther. 41 (7), 636–648. 10.1111/apt.13121 25707624 PMC4654261

[B14] Jenkins H. Jenkins R. Patat A. (2017). Effect of multiple oral doses of the potent CYP3A4 inhibitor clarithromycin on the pharmacokinetics of a single oral dose of vonoprazan: a phase I, open-label, sequential design study. Clin. Drug Invest. 37 (3), 311–316. 10.1007/s40261-016-0488-6 27928738

[B15] Kagami T. Furuta T. (2018). Response to “CYP-mediated drug-drug interaction is not a major determinant of attenuation of antiplatelet function of clopidogrel by vonoprazan”. Clin. Pharmacol. Ther. 104 (1), 33–34. 10.1002/cpt.1059 29663346

[B16] Kagami T. Sahara S. Ichikawa H. Uotani T. Yamade M. Sugimoto M. (2016). Potent acid inhibition by vonoprazan in comparison with esomeprazole, with reference to CYP2C19 genotype. Aliment Pharmacol. Ther. 43 (10), 1048–1059. 10.1111/apt.13588 26991399

[B17] Kagami T. Yamade M. Suzuki T. Uotani T. Hamaya Y. Iwaizumi M. (2018). Comparative study of effects of vonoprazan and esomeprazole on antiplatelet function of clopidogrel or prasugrel in relation to CYP2C19 genotype. Clin. Pharmacol. Ther. 103 (5), 906–913. 10.1002/cpt.863 28875498

[B18] Kawai T. Oda K. Funao N. Nishimura A. Matsumoto Y. Mizokami Y. (2018). Vonoprazan prevents low-dose aspirin-associated ulcer recurrence: randomised phase 3 study. Gut. 67 (6), 1033–1041. 10.1136/gutjnl-2017-314852 29196436 PMC5969345

[B19] Kogame A. Takeuchi T. Nonaka M. Yamasaki H. Kawaguchi N. Bernards A. (2017). Disposition and metabolism of TAK-438 (vonoprazan fumarate), a novel potassium-competitive acid blocker, in rats and dogs. Xenobiotica 47 (3), 255–266. 10.1080/00498254.2016.1182667 27225050

[B20] Ma J. Wang S. Zhang M. Zhang Q. Zhou Y. Lin C. (2015). Simultaneous determination of bupropion, metroprolol, midazolam, phenacetin, omeprazole and tolbutamide in rat plasma by UPLC-MS/MS and its application to cytochrome P450 activity study in rats. BioMed. Chromatogr. 29 (8), 1203–1212. 10.1002/bmc.3409 25582505

[B21] Mizokami Y. Oda K. Funao N. Nishimura A. Soen S. Kawai T. (2018). Vonoprazan prevents ulcer recurrence during long-term NSAID therapy: randomised, lansoprazole-controlled non-inferiority and single-blind extension study. Gut 67 (6), 1042–1051. 10.1136/gutjnl-2017-314010 28988197 PMC5969369

[B22] Nishihara M. Czerniak R. (2018). CYP-mediated drug-drug interaction is not a major determinant of attenuation of antiplatelet function of clopidogrel by vonoprazan. Clin. Pharmacol. Ther. 104 (1), 31–32. 10.1002/cpt.1060 29603200

[B23] Nishihara M. Yamasaki H. Czerniak R. Jenkins H. (2019). In vitro assessment of potential for CYP-inhibition-based drug-drug interaction between vonoprazan and clopidogrel. Eur. J. Drug Metab. Pharmacokinet 44 (2), 217–227. 10.1007/s13318-018-0521-7 30361928

[B24] Nishihara M. (2019). Inhibitory effect of vonoprazan on the metabolism of [^14^C]Prasugrel in human liver microsomes. Eur. J. Drug Metab. Pharmacokinet. 44 (5), 713–717. 10.1007/s13318-019-00554-y 30993551

[B25] O'Connor A. Liou J. M. Gisbert J. P. O'Morain C. (2019). Review: treatment of helicobacter pylori infection 2019. Helicobacter 24 Suppl 1, e12640. 10.1111/hel.12640 31486235

[B26] Rowland A. Mangoni A. A. Hopkins A. Sorich M. J. Rowland A. (2016). Optimized cocktail phenotyping study protocol using physiological based pharmacokinetic modeling and assessment of metabolic drug-drug interactions involving modafinil. Front. Pharmacol. 7 (undefined), 517. 10.3389/fphar.2016.00517 28082902 PMC5186771

[B27] Spechler S. J. Hunter J. G. Jones K. M. Lee R. Smith B. R. Mashimo H. (2019). Randomized trial of medical versus surgical treatment for refractory heartburn. N. Engl. J. Med. 381 (16), 1513–1523. 10.1056/NEJMoa1811424 31618539

[B28] Stoetzer C. Kistner K. Stüber T. Wirths M. Schulze V. Doll T. (2015). Methadone is a local anaesthetic-like inhibitor of neuronal Na+ channels and blocks excitability of mouse peripheral nerves. Br. J. Anaesth 114 (1), 110–120. 10.1093/bja/aeu206 25012584

[B29] Sugimoto M. Yamaoka Y. (2018). Role of vonoprazan in helicobacter pylori eradication therapy in Japan. Front. Pharmacol. 9, 1560. 10.3389/fphar.2018.01560 30697158 PMC6340927

[B30] Takeda (2014). New drug application approval of TAKECAB?for the treatment of acid-related diseases in Japan (media release) [Online]. http://www.takeda.com [Accessed 26 Dec 2014].

[B31] Tansel A. Graham D. Y. (2017). New insight into an effective treatment of marginal ulceration after Roux-en-Y gastric bypass. Clin. Gastroenterol. Hepatol. 15 (4), 501–503. 10.1016/j.cgh.2016.12.025 28062217 PMC6916727

[B32] Tomaz P. R. X. Kajita M. S. Santos J. R. Scholz J. Abe T. O. Gaya P. V. (2019). Cytochrome P450 2A6 and 2B6 polymorphisms and smoking cessation success in patients treated with varenicline. Eur. J. Clin. Pharmacol. 75 (11), 1541–1545. 10.1007/s00228-019-02731-z 31402421

[B33] van Dyk M. Marshall J. C. Sorich M. J. Wood L. S. Rowland A. (2018). Assessment of inter-racial variability in CYP3A4 activity and inducibility among healthy adult males of Caucasian and South Asian ancestries. Eur. J. Clin. Pharmacol. 74 (7), 913–920. 10.1007/s00228-018-2450-4 29572563

[B34] Vazquez S. R. (2018). Drug-drug interactions in an era of multiple anticoagulants: a focus on clinically relevant drug interactions. Blood 132 (21), 2230–2239. 10.1182/blood-2018-06-848747 30463993

[B35] Wang Z. Sun W. Huang C. K. Wang L. Xia M. M. Cui X. (2015). Inhibitory effects of curcumin on activity of cytochrome P450 2C9 enzyme in human and 2C11 in rat liver microsomes. Drug Dev. Ind. Pharm. 41 (4), 613–616. 10.3109/03639045.2014.886697 24517573

[B36] Wilkinson G. R. (2005). Drug metabolism and variability among patients in drug response. N. Engl. J. Med. 352 (21), 2211–2221. 10.1056/NEJMra032424 15917386

[B37] Xiao Y. Zhang S. Dai N. Fei G. Goh K. L. Chun H. J. (2020). Phase III, randomised, double-blind, multicentre study to evaluate the efficacy and safety of vonoprazan compared with lansoprazole in Asian patients with erosive oesophagitis. Gut. 69 (2), 224–230. 10.1136/gutjnl-2019-318365 31409606 PMC6984055

[B38] Xie Y. Bowe B. Yan Y. Xian H. Li T. Al-Aly Z. (2019). Estimates of all cause mortality and cause specific mortality associated with proton pump inhibitors among US veterans: cohort study. BMJ Open Gastroenterol. 365, l1580. 10.1136/bmj.l1580 PMC6538974 31147311

[B39] Yamamoto K. Dubey V. Irie K. Nakanishi H. Khandelia H. Fujiyoshi Y. (2019). A single K-binding site in the crystal structure of the gastric proton pump. Elife 8, undefined. 10.7554/eLife.47701 PMC6706254 31436534

[B40] Yamasaki H. Kawaguchi N. Nonaka M. Takahashi J. Morohashi A. Hirabayashi H. (2017). In vitro metabolism of TAK-438, vonoprazan fumarate, a novel potassium-competitive acid blocker. Xenobiotica 47 (12), 1027–1034. 10.1080/00498254.2016.1203505 27414183

[B41] Yamasaki A. Yoshio T. Muramatsu Y. Horiuchi Y. Ishiyama A. Hirasawa T. (2018). Vonoprazan is superior to rabeprazole for healing endoscopic submucosal dissection: induced ulcers. Digestion 97 (2), 170–176. 10.1159/000485028 29310111

[B42] Yasumuro O. Uchida S. Kashiwagura Y. Suzuki A. Tanaka S. Inui N. (2018). Changes in gefitinib, erlotinib and osimertinib pharmacokinetics under various gastric pH levels following oral administration of omeprazole and vonoprazan in rats. Xenobiotica 48 (11), 1106–1112. 10.1080/00498254.2017.1396379 29057719

